# Supplementary material for: Contribution of individual olfactory receptors to odor-induced attractive or aversive behavior in mice
Source: Nat Commun. 2019 Jan 14;10:209. doi: 10.1038/s41467-018-07940-1 (PMC6331590; doi:10.1038/s41467-018-07940-1)
Supplement: Supplementary file 1 — Supplementary Information [file 41467_2018_7940_MOESM1_ESM.pdf]

Supplementary Information

Contribution of individual olfactory receptors to odor-induced attractive or aversive behavior in mice

Horio et al.,

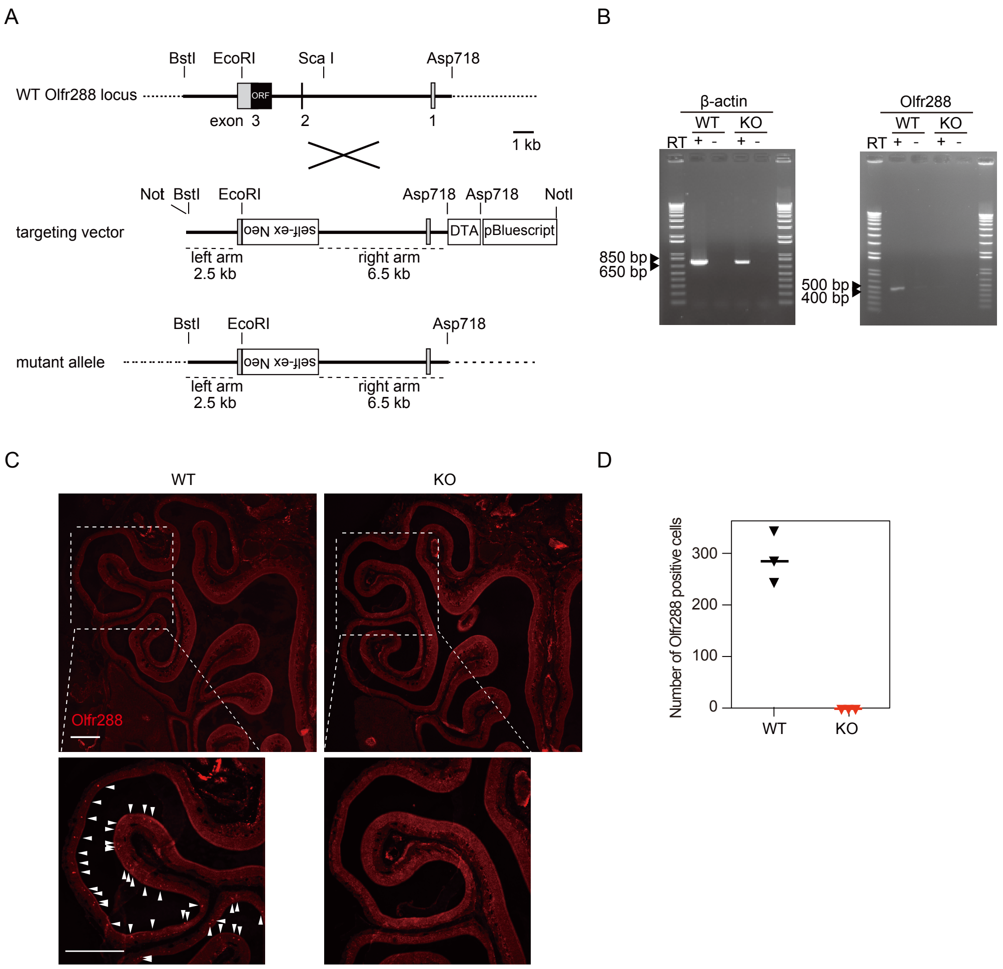

Supplementary Figure 1

Expressions of Olfr288 in the olfactory epithelium

(A) Schematic representation showing the structure of the Olfr288 gene and the strategy for generating knock-out mice. Neo: neomycin resistant gene; DTA: diphtheria toxin A-chain gene

(B) mRNA expression of β-actin and Olfr288 in the main olfactory epithelium of C57BL/6 adult mice (WT) and Olfr288-KO adult mice (KO) mice by RT-PCR.

(C) Fluorescent staining of the main olfactory epithelium sections labeled with Olfr288 cRNA probe in C57BL/6 adult mice (WT, left) and Olfr288-KO adult mice (KO, right) mice. Scale bars, 300 μm.

(D) Number of Olfr288 positive cells in four coronal sections from WT and KO (N=3 each) mice.

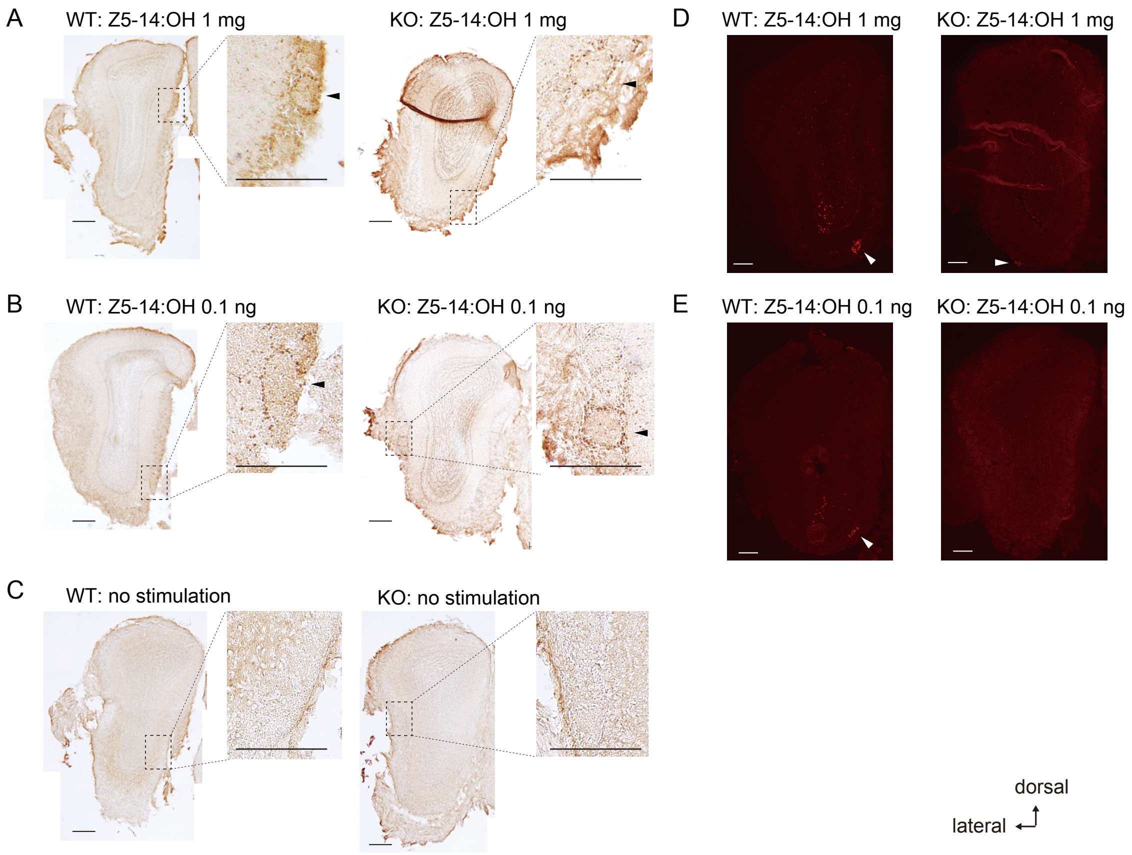

Supplementary Figure 2

Z5-14:OH-evoked c-Fos expression in the OB of WT and Olfr288-KO mice

(A-C) Representative c-Fos expression by immunohistochemistry in the OB sections of C57BL/6 adult female mice (WT) and Olfr288-KO adult female mice (KO) stimulated with 1 mg Z5-14:OH (A), 0.1 ng Z5-14:OH (B) or no odor (C). c-Fos, an immediate early gene product, was used as an indicator of glomerular activation to examine responses to Z5-14:OH in the OB. Right panels in each OB section show enlarged views of the region indicated by the dashed square in the left panels. Arrowheads indicate activated glomeruli surrounded by c-Fos-positive juxtaglomerular cells. Scale bars, 200 μm.

(D, E) Representative c-Fos expression by in situ hybridization in the OB sections of C57BL/6 adult female mice (WT) and Olfr288-KO adult female mice (KO) stimulated with 1 mg Z5-14:OH (D) or 0.1 ng Z5-14:OH (E). Arrowheads indicate activated glomeruli surrounded by c-Fos-positive juxtaglomerular cells. Scale bars, 200 μm.

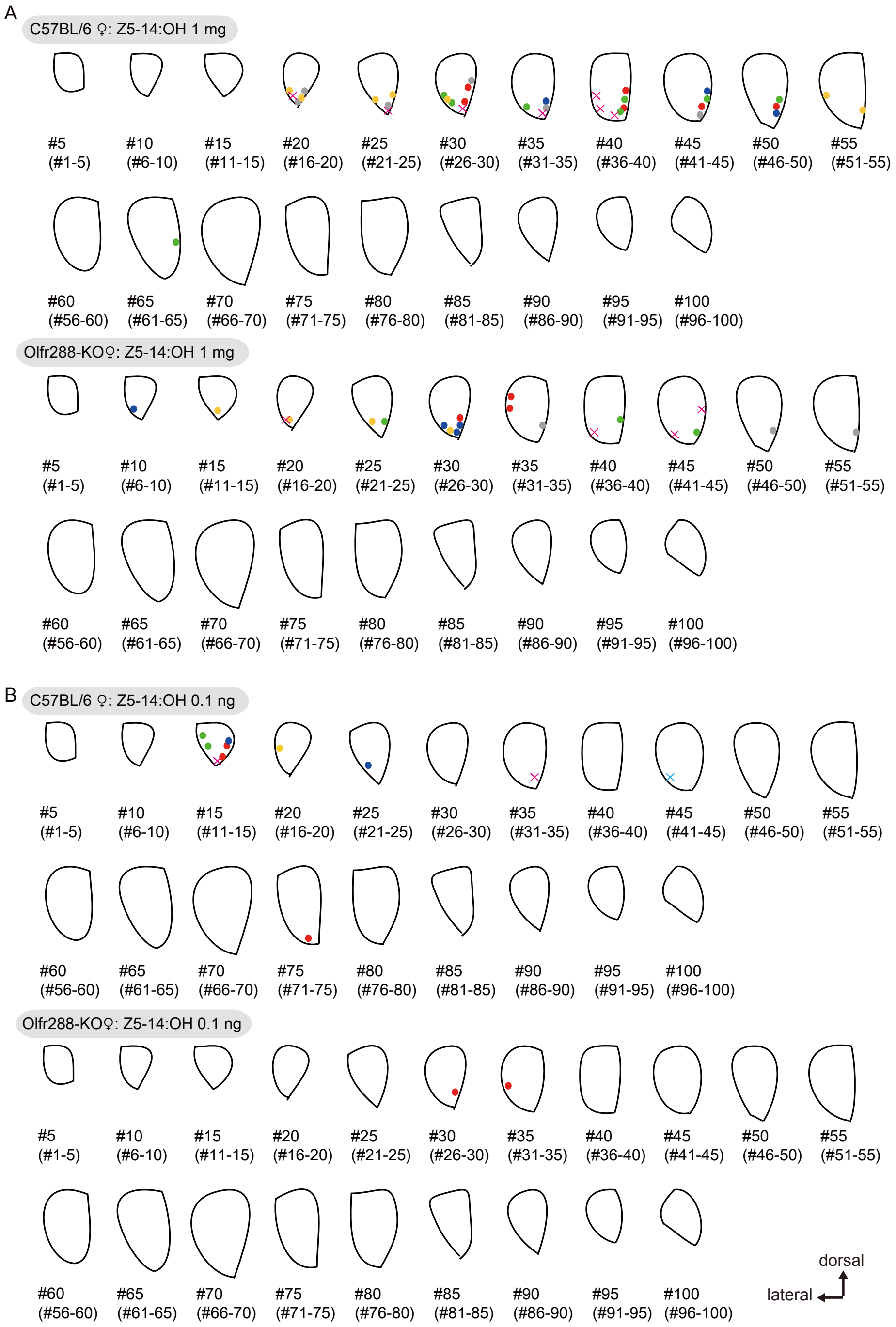

Supplementary Figure 3

Distribution of glomeruli responding to Z5-14:OH

(A, B) Summary of the positional distribution of glomeruli activated by 1 mg (A) and 0.1 ng (B) Z5-14:OH in C57BL/6 adult female mice (WT) and Olfr288-KO adult female mice (KO) in Figure 2A. Each schematic drawing includes serial 5 sections from the anterior tip of the OB. #1 is the anterior tip of the OB. The same color in each schematic drawing indicates the same animal. Each circle symbol depicts the result of immunohistochemistry and each cross symbol depicts the result of in situ hybridization.

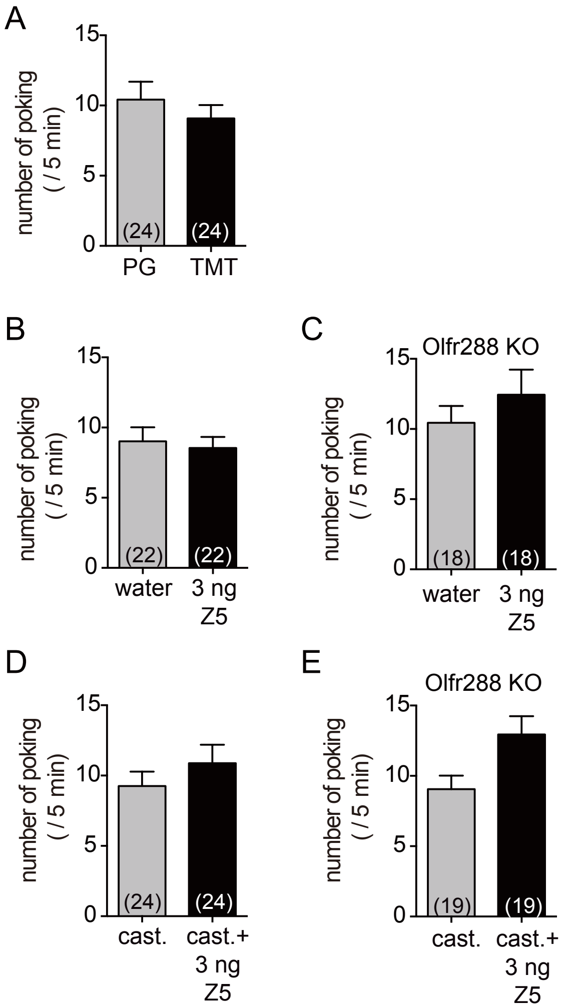

Supplementary Figure 4

Number of poking a hole of Z5-14:OH in the two-choice odor-preference test

(A) Number of poking a nose into holes in the control experiments in the two-choice odor-preference test in Figure 3.

(B, C) Number of poking a nose to 3 ng Z5-14:OH in adult female C57BL/6 mice (B) and adult female Olfr288-KO mice (C) in Figure 3. Z5: Z5-14:OH

(D, E) Number of poking a nose to castrated male urine with or without 3 ng Z5-14:OH, in adult female C57BL/6 mice (D) and adult female Olfr288-KO mice (E).

Bars indicate the number of poking a nose to each test sample within a 3-min period, ± S.E.M.. There was no significant difference between two samples in all figures (paired Student's t-test).

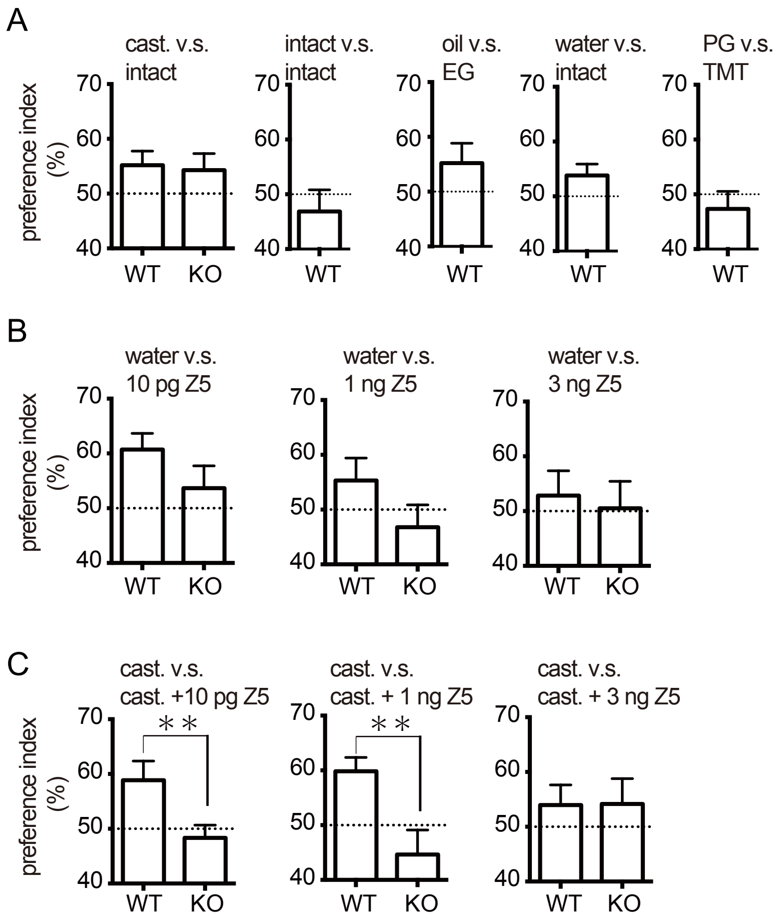

Supplementary Figure 5

Preference index to Z5-14:OH

(A) Preference index to a series of control experiments of adult female C57BL/6 mice (WT) and adult female Olfr288-KO mice (KO) in the two-choice odor-preference test in Figure 3. Preference index means the ratio of investigation time of poking a nose into a hole with a targeted odorant to the total investigation time into both holes. Intact: urine from intact male C57BL/6 mice; cast.: urine from castrated male C57BL/6 mice. oil: mineral oil; EG: eugenol.

(B) Preference index to various amounts of Z5-14:OH of adult female C57BL/6 mice (WT) and adult female Olfr288-KO mice (KO).

(C) Preference index to castrated male urine with or without 3 ng Z5-14:OH, of adult female C57BL/6 mice (WT) and adult female Olfr288-KO mice (KO).

Data are means ± S.E.M. Mann–Whitney test: $^{**}P<0.01$
